# Coinfection of viruses in children with community-acquired pneumonia

**DOI:** 10.1186/s12887-024-04939-0

**Published:** 2024-07-16

**Authors:** Song Mao, Liangxia Wu

**Affiliations:** Department of Pediatrics, Shanghai Sixth People’s Hospital, Affiliated to Shanghai Jiao Tong University School of Medicine, Shanghai, China

**Keywords:** Virus, Community-acquired pneumonia, Coinfection, Children

## Abstract

**Background:**

Virus, particularly respiratory tract virus infection is likely to co-occur in children with community-acquired pneumonia (CAP). Study focusing on the association between common viruses coinfection and children with CAP is rare. We aimed to study the association between seven common viruses coinfection and clinical/laboratory indexes in children with CAP.

**Methods:**

Six hundred and eighty-four CAP cases from our hospital were enrolled retrospectively. Seven common viruses, including influenza A (FluA), influenza B (FluB), human parainfluenza virus (HPIV), Esptein-Barr virus (EBV), coxsackie virus (CoxsV), cytomegalovirus (CMV), and herpes simplex virus (HSV) were investigated for their associations with CAP. We analyzed the differences of hospitalization days, white blood cell (WBC), c-reactive protein (CRP), platelet (PLT), erythrocyte sedimentation rate (ESR), procalcitonin (PCT), urine red blood cell (uRBC), blood urea nitrogen (BUN), serum creatinine (Scr), alanine aminotransferase (ALT), aspartate aminotransferase (AST), lactate dehydrogenase (LDH), creatine kinase (CK) and creatine kinase isoenzyme (CKMB) among different viruses coinfection groups by using one-way ANOVA analysis. The differences of clinical/laboratory indexes between ordinary and severe pneumonia groups, as well as non-virus vs multi co-infection viruses groups, and single vs multi co-infection viruses groups by using independent samples T test. Receiver operating characteristic (ROC) curve analyses were applied to test the the predictive value of the clinical/laboratory parameters for the risk of viruses coinfections among CAP. Binary logistic analysis was performed to test the association between various indexes and viruses co-infection.

**Results:**

Eighty-four multiple viruses coinfections yielded different prognosis compared with that in 220 single virus coinfection. CMV coinfection was associated with longest hospitalization days, highest ALT, AST and CKMB level. HSV coinfection was associated with highest WBC count, CRP, ESR, and BUN. EBV coinfection was associated with highest PLT and PCT level. FluB coinfection was associated with highest Scr level. CoxsV coinfection was associated with highest uRBC, LDH and CK level. ROC curve analyses showed that CK had the largest area under the curve (AUC: 0.672, p < 10^–4^) for the risk of viruses coinfections risk in CAP. Significant association between PLT, uRBC, BUN, CK, and CKMB and virus coinfection risk in CAP was observed.

**Conclusions:**

Multiple viruses coinfections indicated different prognosis. Different viruses coinfection yielded varying degrees of effects on the cardiac, liver, kidney and inflamatory injury in CAP. The alterations of clinical/laboratory parameters, particularly CK may be associated with the risk of viruses coinfections in CAP.

**Supplementary Information:**

The online version contains supplementary material available at 10.1186/s12887-024-04939-0.

## Introduction

Viruses infection is one of the most common health problems across the world [1]. CAP is likely to occur in the low immunity people, such as the aged and children in the cold season. Environmental pollution, crowd gathering, communication, and viruses coinfection may promote the onset of CAP, even leading to the injury of multi-organs. CAP accounts for the most common cause of death in children less than 5 years old [2]. On the other hand, many children are hospitalized due to CAP, particularly during the epidemic season, which leads to enormous social and economic burden. Hence, early prevention and treatment of CAP seems of great implications.

Streptococcus is the most common pathogen of CAP, appropriate antibiotic use yielded good therapeutic effects [3]. Mycoplasma pneumoniae is another common cause of atypical pneumonia presenting with its different course and extrapulmonary complications [4]. Azithromycin use shows good effects on mycoplasma pneumonia [5]. Active surveillance of these pathogens is helpful for the treatment of CAP. Notably, a number of CAP cases were refractory to conventional therapy, viruses coinfections may be an important cause, which affects the prognosis of CAP. Viruses are also the most commonly detected pathogens in children with CAP [6]. Respiratory syncytial virus (RSV) and rhinovirus were the most commonly detected causes in pediatric pneumonia [7]. RSV is likely to induce asthma-like symptoms, enterovirus is prone to lead to the onset of rash [8, 9]. On the other hand, the epidemic COVID-19 may also usually induce the lung injury, exacerbating the lung function [10]. Some severe CAP cases are even complicated with more than two viruses coinfections, leading to serious symptoms. Therefore, an in-depth understanding regarding the viruses coinfection during the course of CAP was of great significances.

Recently, etiological studies of children with CAP focused on the distribution of viruses in different ethnics, regions, populations and seasons. For example, RSV is commonly observed in the lower aged children [11]. Coinfections of multiple pathogens lead to more severe symptoms [12]. However, the specific association of CAP clinical/laboratory indexes with viruses coinfection was rare. We performed this retrospective study to analyse deeply the incidence, characteristics and differences of seven common viruses coinfections in children with CAP. We also determined the impact of seven common viruses coinfections on the clinical and laboratory parameters, including the hospitalisation days, inflammatory indexes, cardiac injury indexes, liver injury indexes, and kidney injury indexes. Additionally, receiver operating characteristic (ROC) curve analysis was used to calculate the predictive value of these parameters in the risk of certain viruses coinfections in CAP.

## Methods

### Study design

A retrospective investigation of seven common viruses coinfections in children with CAP was performed. This study was conducted to yield the impact of these viruses coinfections on the children with CAP. The study was approved by ethics committee of Shanghai Sixth People’s Hospital (Registration Clinical Trial Number. 2018–106). This study was performed according to the Declaration of Helsinki. As the study was performed in the retrospective style, the informed consent of guardians of participants was waived. All the included data was de-identified. We conducted the anonymous data analysis in the retrospective style.

### Patient population

All the enrolled cases were the patients admitted to the Department of Pediatrics, Shanghai Sixth People’s Hospital, China. The age was between 1 and 14 years. The study period was between June 2016 and June 2018. The patients with systemic diseases that may affect the prognosis of CAP were excluded. All the recruited cases were inpatients.

### Data collection

We extracted the clinical and laboratory parameters from the electronical medical records. We collected the data of age, gender, infected viruses and hospitalization days. In the meantime, we also collected the laboratory data, including white blood cell (WBC), c-reactive protein (CRP), platelet (PLT), erythrocyte sedimentation rate (ESR), procalcitonin (PCT), urine red blood cell (uRBC),

blood urea nitrogen (BUN), serum creatinine (Scr), alanine aminotransferase (ALT), Aspartate aminotransferase (AST), lactate dehydrogenase (LDH), creatine kinase (CK) and creatine kinase isoenzyme (CKMB). All the laboratory parameters were tested within three days after admission. CAP was divided into two types: ordinary pneumonia (OP) and severe pneumonia (SP). SP was defined as that multiple systems were injured besides the lung (presence of danger signs: persistent vomiting, lethargy or unconsciousness, seizures, severe malnutrition, stridor in a calm child or not able to drink) [13].

### Virus detection

The panel for virus detection was done before the collection of specimens for biomarker analysis.

The nasopharyngeal swabs were collected for testing influenza A (FluA), influenza B (FluB), human parainfluenza virus (HPIV). Multiplex PCR assay was used for the detection of FluA, Flu B, and HPIV. The sensitivity of the multiplex PCR was evaluated by testing the limit of detection of the assay. The specificity of the multiplex PCR assay was evaluated by cross reaction tests with other common respiratory pathogens. We have no false positive results. Plasma samples were collected for testing Esptein-Barr virus (EBV), coxsackie virus (CoxsV), cytomegalovirus (CMV), and herpes simplex virus (HSV). ELISA method was used for the detection of IgM antibodies levels of EBV, CoxsV, CMV, and HSV. The samples were handled according to the manufacturer’s instructions. The tests were performed in the clinical laboratory of Shanghai Sixth People’s Hospital. Virus detection was performed after the collection of specimens. The results were defined as positive or negative with no quantitative analyses. The results were collected retrospectively.

### Statistical analysis

Continuous variables were expressed as means ± standard deviation (SD), while categorical variables were expressed as percentages. Descriptive analyze were conducted to investigate the differences of these indexes among various viruses coinfection groups. Upset plot was used to show the distribution of multi viruses coinfections. One-way ANOVA analysis was applied to test the differences of these indexes among non-virus, single virus and multiple viruses coinfections groups. Independent samples T test was to determine the differences of these indexes between OP and SP groups, as well as non-virus vs multi co-infection viruses groups, and single vs multi co-infection viruses groups. ROC curve analysis was applied to test the predictive value of these parameters for the risk of virus coinfections among CAP. The Youden index is equal to sensitivity + 1-specificity. The corresponding point of Youden index is regarded as the optimal cut-off point. The ROC area > 0.50 with *p* value < 0.05 was regarded as the significant difference. Binary logistic analysis was performed to test the association between various indexes and viruses co-infection. All the quantitative analyses were performed by using SPSS version 19. *P* < 0.05 was considered statistically significant, except where otherwise specified.

## Results

### Incidence and distribution of virus

A total of 684 CAP cases were enrolled in our study. Among the recruited cases, 540 were OP, and 144 were SP. 380 cases were not infected with any virus, 220 cases were infected with single virus, and 84 cases were infected with at least two viruses. There are 42 FluA cases, 50 Flu B cases, 20 HPIV cases, 36 HSV cases, 108 EBV cases, 32 CMV cases, and 16 CoxsV cases (Table 1). Upset plot showed the distribution of multi viruses co-infections (Supplemental material 1).

**Table 1:** Baseline characteristics of enrolled participants

Groups	N	Age (years)	Male number/male percentage
Type of disease			
Ordinary pneumonia	540	4.65 ± 2.52	292/54.07%
Severe pneumonia	144	3.85 ± 2.19	88/61.1%
Type of virus infected			
Influenza A virus	42	4.67 ± 1.74	24/57.1%
Influenza B virus	50	7.00 ± 3.41	20/40%
HPIV	20	5.60 ± 1.05	12/60%
HSV	36	3.67 ± 2.11	21/58.3%
EBV	108	3.40 ± 1.79	58/53.7%
CMV	32	2.00 ± 1.03	17/53.1%
CoxsV	16	2.50 ± 1.71	8/50%
Non-virus	380	4.86 ± 2.58	180/47.4%
Single virus infected	220	4.27 ± 2.49	125/56.9%
Multiple viruses infected	84	4.58 ± 2.55	37/44.0%

### Descriptive analyses of various indexes among cases with viruses coinfections

CMV, EBV, and CoxsV coinfections were associated with the longest hospitalization days (9.00 ± 3.05, 7.87 ± 1.90, and 7.75 ± 2.46, respectively). HSV coinfection was associated with the shortest hospitalization days (5.50 ± 1.53). HSV, CMV, and HPIV coinfections were associated with the highest WBC count (15.93 ± 4.40, 12.80 ± 4.06, and 8.54 ± 3.97, respectively). FluA virus was associated with lowest WBC count (6.33 ± 2.95). HSV, CMV, and EBV coinfections were associated with the highest CRP level (63.67 ± 39.96, 48.35 ± 46.58, and 23.76 ± 36.55, respectively). FluB coinfection was associated with the lowest CRP level (8.64 ± 10.04). EBV, HSV and FluB coinfections were associated with the highest PLT level (283.11 ± 181.52, 275.33 ± 30.88, and 278.20 ± 125.98, respectively).

FluA coinfection was associated with the lowest PLT level (220.66 ± 52.61). HSV, FluA and EBV coinfections were associated with the highest ESR level (38.67 ± 22.01, 27.00 ± 10.81, and 22.30 ± 10.96, respectively). HPIV coinfection was associated with the lowest ESR level (12.80 ± 7.72).

EBV, HSV and FluA coinfections were associated with the highest PCT level (1.70 ± 5.06, 1.51 ± 1.32, and 0.57 ± 0.39, respectively). HPIV coinfection was associated with the lowest PCT level (0.08 ± 0.01). CoxsV, HSV and EBV coinfections were associated with the highest uRBC level (10.00 ± 5.89, 10.00 ± 4.38, and 8.72 ± 9.53, respectively). FluA coinfection was associated with the lowest uRBC level (2.00 ± 1.65). HSV, HPIV and CoxsV coinfections were associated with the highest BUN level (4.07 ± 0.17, 3.82 ± 0.89, and 3.57 ± 1.27, respectively). CMV coinfection was associated with the lowest BUN level (2.80 ± 0.20). FluB, FluA and HPIV coinfections were associated with the highest Scr level (33.80 ± 7.87, 30.00 ± 11.48, and 27.40 ± 4.57, respectively). CMV coinfection was associated with the lowest Scr level (16.00 ± 0.01). CMV, HSV and CoxsV coinfections were associated with the highest ALT level (229.00 ± 203.20, 38.33 ± 32.10, and 17.25 ± 9.39, respectively). HPIV infection was associated with the lowest ALT level (9.20 ± 1.36). CMV, HSV and CoxsV coinfections were associated with the highest AST level (206.50 ± 169.16, 34.33 ± 9.70, and 32.00 ± 2.63, respectively). HPIV coinfection was associated with the lowest AST level (24.20 ± 2.71). CoxsV, CMV and HSV coinfections were associated with the highest LDH level (401.00 ± 143.17, 353.00 ± 51.82, and 301.66 ± 30.64, respectively). FluA coinfection was associated with the lowest LDH level (242.33 ± 24.38). CoxsV, FluB and HSV coinfections were associated with the highest CK level (56.77 ± 16.07, 128.00 ± 76.02, and 93.67 ± 27.33, respectively). FluA coinfection was associated with the lowest CK level (59.67 ± 7.45). CMV, CoxsV and HSV coinfections were associated with the highest CKMB level (34.00 ± 5.08, 31.00 ± 1.48, and 28.33 ± 11.63, respectively). FluA coinfection was associated with the lowest CKMB level (15.00 ± 5.78) (Table 2, Supplemental material 2).

**Table 2:** Baseline characteristics of indexes among cases

	FluA	FluB	HPIV	HSV	EBV	CMV	CoxsV
Days	6.00 (0.83)	6.60 (0.81)	7.00 (0.65)	5.50 (1.53)	7.87 (1.90)	9.00 (3.05)	7.75 (2.46)
WBC	6.33 (2.95)	6.74 (3.12)	8.54 (3.97)	15.93 (4.40)	6.83 (3.05)	12.80 (4.06)	6.73 (2.81)
CRP	13.09 (5.88)	8.64 (10.04)	11.14 (10.61)	63.67 (39.96)	23.76 (36.55)	48.35 (46.58)	11.46 (15.26)
PLT	220.66 (52.61)	278.20 (125.98)	234.40 (50.72)	275.33 (30.88)	283.11 (181.52)	244.00 (8.13)	256.25 (62.32)
ESR	27.00 (10.81)	22.10 (17.32)	12.80 (7.72)	38.67 (22.01)	22.30 (10.96)	21.50 (19.81)	15.50 (62.32)
PCT	0.57 (0.39)	0.18 (0.16)	0.08 (0.01)	1.51 (1.32)	1.70 (5.06)	0.32 (0.22)	0.33 (0.17)
uRBC	2.00 (1.65)	3.50 (2.48)	6.20 (3.97)	10.00 (4.38)	8.72 (9.53)	5.50 (3.56)	10.00 (5.89)
BUN	3.00 (0.38)	3.28 (1.45)	3.82 (0.89)	4.07 (0.17)	3.26 (0.89)	2.80 (0.20)	3.57 (1.27)
Scr	30.00 (11.48)	33.80 (7.87)	27.40 (4.57)	27.67 (5.06)	26.80 (6.46)	16.00 (0.01)	23.50 (9.54)
ALT	10.33 (3.13)	13.00 (6.84)	9.20 (1.36)	38.33 (32.10)	16.93 (9.73)	229.00 (203.20)	17.25 (9.39)
AST	26.67 (5.63)	28.70 (10.66)	24.20 (2.71)	34.33 (9.70)	31.66 (8.44)	206.50 (169.16)	32.00 (2.63)
LDH	242.33 (24.38)	255.80 (44.58)	250.40 (35.75)	301.66 (30.64)	289.61 (33.81)	353.00 (51.82)	401.00 (143.17)
CK	59.67 (7.45)	128.00 (76.02)	67.00 (30.41)	93.67 (27.33)	72.75 (25.01)	76.50 (3.56)	146.00 (29.03)
CKMB	15.00 (5.78)	20.70 (5.50)	17.60 (4.52)	28.33 (11.63)	21.75 (6.92)	34.00 (5.08)	31.00 (1.48)

Days (n), WBC (109/L), PLT(109/L), ESR(mm/hr), PCT(ng/ml), uRBC(n/ul), BUN(mmol/L), Scr(umol/L), CRP(umol/L), LDH(mmol/L), CK(mmol/L), CKMB(mmol/L), ALT(u/L), AST(u/L)

### Distribution of various viruses, and differences of various parameters among OP and SP

A total of 540 OP cases were enrolled. There were 74 EBV cases, 29 HSV cases, 16 HPIV cases, 36 FluA cases, 40 FluB cases, 22 CMV cases, and 12 CoxsV cases, respectively. A total of 144 SP cases were recruited. There were 34 EBV cases, 7 HSV cases, 4 HPIV cases, 6 FluA cases, 10 FluB cases, 10 CMV cases, and 4 CoxsV cases, respectively.

There were significant differences of days, WBC, CRP, PCT, uRBC, BUN, LDH, and CKMB between OP and SP. No marked differences of PLT, ESR, Scr, ALT, AST, and CK were noted between OP and SP (Table 3).

**Table 3:** Differences of various indexes between OP and SP

Index	OP	SP	P (OP vs SP)
			Independent samples T test
Days	6.91 (1.81)	8.44 (2.04)	< 0.0001
WBC	7.50 (5.52)	12.06 (7.11)	< 0.0001
CRP	20.32 (33.72)	30.62 (38.91)	0.002
PLT	245.46 (111.96)	265.05 (89.80)	0.053
ESR	20.82 (19.57)	20.67 (15.32)	0.930
PCT	0.52 (1.83)	1.07 (1.54)	0.002
uRBC	5.74 (6.90)	8.89 (8.72)	< 0.0001
BUN	3.34 (0.97)	3.57 (1.33)	0.024
Scr	28.88 (8.00)	30.17 (9.95)	0.108
ALT	18.14 (37.52)	17.72 (10.66)	0.895
AST	34.85 (31.81)	35.50 (6.64)	0.806
LDH	289.90 (76.17)	360.27 (162.83)	< 0.0001
CK	127.19 (131.79)	113.73 (58.65)	0.275
CKMB	23.32 (9.01)	20.93 (6.03)	0.006

*OP* ordinary pneumonia, *SP* severe pneumonia

### Differences of various parameters among non-virus, single virus and multiple viruses

Significant differences of days, WBC, CRP, PLT, ESR, PCT, uRBC, BUN, ALT, AST, LDH, CK, and CKMB among non-virus, single virus and multiple viruses were observed. No marked differences of Scr were noted among non-virus, single virus and multiple viruses (Table 4). Significant differences of WBC, CRP, PLT, ESR, BUN, LDH, CK, and CKMB between non-virus and multiple viruses were observed. No marked differences of days, PCT, uRBC, Scr, ALT, and AST were noted between non-virus and multiple viruses (Table 4). Significant differences of WBC, CRP, PLT, ALT, AST, and LDH were observed between single and multiple viruses were observed. No marked differences of days, PLT, ESR, PCT, uRBC, BUN, Scr, CK, and CKMB were noted between single and multiple viruses (Table 4).

**Table 4:** Differences of various indexes among non, single and multiviruses infections

Index	Non	Sing	Mult	P (Non vs Sing vs Mult)	P (Non vs Mult)	P (Sing vs Mult)
				One-way ANOVA analysis	Independent samples T test	
Days	6.72 (1.78)	7.17 (1.75)	7.08 (2.05)	0.011	0.139	0.725
WBC	6.64 (5.98)	8.60 (7.49)	10.95 (5.21)	< 0.0001	< 0.0001	0.010
CRP	16.40 (29.30)	22.29 (33.04)	36.58 (52.19)	< 0.0001	0.001	0.005
PLT	226.64 (92.55)	268.97 (135.38)	284.25 (118.77)	< 0.0001	< 0.0001	0.328
ESR	19.30 (20.91)	22.32 (16.34)	25.75 (21.43)	0.016	0.012	0.167
PCT	0.36 (0.81)	0.86 (2.93)	0.48 (0.76)	0.005	0.205	0.077
uRBC	5.16 (6.90)	6.81 (6.91)	5.11 (5.71)	0.017	0.950	0.053
BUN	3.22 (0.98)	3.42 (1.02)	3.62 (0.65)	0.001	< 0.0001	0.096
Scr	29.25 (7.90)	2.78 (7.39)	29.60 (10.78)	0.059	0.785	0.086
ALT	14.69 (6.68)	16.14 (13.07)	31.89 (89.50)	< 0.0001	0.082	0.013
AST	33.83 (11.83)	29.93 (8.35)	46.10 (74.22)	< 0.0001	0.135	0.002
LDH	297.09 (76.04)	286.60 (67.07)	261.06 (84.23)	< 0.0001	< 0.0001	0.013
CK	56.77 (16.07)	88.26 (38.79)	91.19 (54.99)	< 0.0001	< 0.0001	0.690
CKMB	24.34 (10.06)	21.76 (7.99)	21.38 (6.13)	0.001	0.001	0.640

*Non* non-virus, *Sing* single virus, *Mult* multiple viruses

### ROC curve analysis of the predictive value of various indexes in virus coinfections of CAP

Significant association between the parameters of ALT, AST, LDH, CK, and CKMB and virus coinfection in CAP was observed. The optimal cut-off points and sensitivity and specificity for parameters mentioned above are shown in Table 5.

**Table 5:** Association between various indexes and virus infection

Index	Sensitivity	Specificity	ROC area	95% CI	*P*	Cut-off point	*P*	Exp(B) 95%CI	Logistic regression	
Days	0.264	0.654	0.439	0.395–0.483	0.007	7.50	0.271	1.061(0.955–1.180)	7.50	2.33
WBC	0.274	0.474	0.328	0.287–0.370	< 0.0001	7.05	0.185	1.028 (0.987–1.072)	7.05	2.32
CRP	0.288	0.586	0.407	0.364–0.451	< 0.0001	12.17	0.086	0.992(0.984–1.001)	12.17	2.33
PLT	0.397	0.394	0.381	0.339–0.424	< 0.0001	227.00	< 0.0001	1.005(1.003–1.008)	227.00	
ESR	0.257	0.643	0.390	0.347–0.434	< 0.0001	24.50	0.197	0.991(0.978–1.005)	24.50	2.29
PCT	0.400	0.460	0.417	0.373–0.460	< 0.0001	0.16	0.901	1.007(0.899–1.129)	0.16	
uRBC	0.118	0.838	0.413	0.370–0.456	< 0.0001	11.50	0.040	1.030(1.001–1.060)	11.50	
BUN	0.310	0.530	0.406	0.363–0.450	< 0.0001	3.45	< 0.0001	1.688(1.365–2.087)	3.45	
Scr	0.451	0.530	0.524	0.479–0.569	0.284	29.50	0.062	0.975(0.950–1.001)	29.50	
ALT	0.141	0.808	0.567	0.522–0.612	0.003	20.50	0.867	1.003(0.966–1.042)	20.50	
AST	0.479	0.653	0.584	0.540–0.627	< 0.0001	33.50	0.057	1.036(0.999–1.074)	33.50	
LDH	0.535	0.593	0.589	0.545–0.633	< 0.0001	276.50	0.057	0.996(0.992–1.000)	276.50	
CK	0.557	0.699	0.672	0.630–0.713	< 0.0001	102.50	< 0.0001	0.991(0.986–0.995)	102.50	
CKMB	0.457	0.688	0.578	0.534–0.623	0.001	24.50	0.006	0.961(0.935–0.989)	24.50	

*ROC* receiver operating characteristic curve

### Binary logistic regression analysis of the association between various indexes and virus coinfections of CAP

Significant association between PLT, uRBC, BUN, CK, and CKMB and virus coinfection in CAP was observed (Table 5).

## Discussion

Viruses coinfections are important facilitators of CAP susceptibility and progression [14]. Respiratory viruses are frequently detected in CAP among children. RSV, rhinovirus, and HMPV were the viruses most commonly detected in CAP. Moreover, many other viruses may also influence the development and progression of CAP. Notably, COVID-19 did not primarily manifest as CAP in immunocompetent children [15]. Hence, identification of the detailed role of other common viruses in children with CAP have important clinical implications.

Our study investigated the association between seven common viruses and children with CAP, which is of great implications for pediatric health. We found that multiple viruses coinfections resulted in different prognosis compared with those in non-virus and single virus coinfections. Different viruses coinfection yielded varying degrees of effects on the cardiac, liver, kidney and inflamatory injury in CAP. EBV coinfection was the most common virus detected in CAP. FluB and CMV were the second most common infected virus among OP and SP, respectively. Upset plot also indicated that EBV and FluB were the most common co-infected viruses. The status of clinical and laboratory parameters may be associated with viruses coinfections risk in CAP. Our findings were of great implications that specific clincial characteristics may indicate certain virus coinfection, monitoring of susceptible viruses in specific CAP cases will be helpful for the therapy of CAP in children, and different virus infection may be likely to be associated with different clinical presentations.

Several facts may explain our findings. First: EBV coinfection is likely occur early in life [16]. Many EBV coinfection cases are almost asymptomatic, meriting little attention, leading to the lower testing of EBV. Reactivation of EBV coinfection occurs in the low-immunity state [17], which accounted for the higher incidence of EBV coinfection in CAP. Although EBV is traditionally regarded as with no serious impact on the children, we found that EBV coinfection was associated higher level of PCT and PLT, which indicated that EBV coinfection may also affect the inflammatory state. FluB and CMV were the second most common infected virus among OP and SP, respectively. FluB virus is the common virus during the epidemic season [18]. FluB virus coinfection did not usually lead to serious problems, which explained the higher incidence of FluB virus coinfection in CAP. CMV coinfection was likely to lead to the refractory inflammation and liver injury, which explained that the higher incidence of CMV coinfection in SP [19]. On the other hand, we observed that CMV coinfection was associated with longest hospitalization days, highest ALT, AST and CKMB level, which indicated that CMV may lead to the poor prognosis, including the cardiac and liver injury. Monitoring of CMV coinfection may be needed among the the CAP cases with poor clinical presentations. We also noted that HSV coinfection was associated with highest WBC count, CRP level, ESR, and BUN levels, which suggested that HSV coinfection may aggravate the inflammatory state. HSV may affect the immune response, leading to the inflammation [20]. CoxsV coinfection was associated with highest uRBC, LDH and CK level, which indicated that CoxsV virus coinfection was closely associated with kidney and cardiac injury. Myocardial cells were prone to be injured by CoxsV coinfection [21].

Another important finding was that ROC curve analysis showed that CK had the largest ROC area under the curve for the risk of viruses coinfections in CAP. Binary logistic regression analysis also showed a significant association between CK and virus coinfection risk in CAP. Increased level of CK was closely associated with the viruses coninfection. This observation was of great implications that we should pay more attention to the possibility of viruses coinfection while the CK was increased markedly. Virus coinfections affect the inflammatory state, cardiac function, liver function, and kidney function, which may be due to that virus was likely to injure the tissues [22]. Therefore, monitoring of various parameters may be helpful for the prevention and therapy of virus coinfections.

Our study has important clinical implications that specific virus coinfection affects the progression of CAP. We also investigated some non-respiratory viruses, including EBV, CoxsV, CMV and HSV, which further deepen our knowledge regarding the association between CAP and various non-respiratory viruses. CK may be associated with the risk of viruses coinfection in CAP. We found that significant differences of parameters of cardiac injury, liver injury, kidney injury and inflammaton existed among different virus-coinfected populations. Hence, alteration of certain indexes may reflect the severity of certain coinfected virus, which is helpful for the effective early virus test. Meanwhile, several limitations should be considered in our study. First, the interaction between viruses and other pathogens may affect the prognosis of CAP. Comprehensive analysis of the interaction between viruses and other pathogens should be performed in the future. Second, further studies should be performed to clarify the detailed association between parameters and virus coinfection, such as the relatonship between alterations of biochemical indexes and viral load. Third, the immune status may affect the susceptibility to various viruses infections. Host genetic factors may play an important role in the outcome of respiratory tract infections. Previous study showed that two complement-related SNPs, rare TT genotype of CD55 and rare AA genotype of C1QBP, were associated with increased death risk of influenza, which indicated that mutations related to immunity may affect the prognosis [23]. We need to consider the host genetic factors while investigating the association between viruses infections and CAP in the future.

Finally, a prospective study design should be applied by excluding the confounding factors in the future. The data were extracted from June 2016 to June 2018 before the outbreak of COVID-19, we did not study the association between COVID-19 and CAP. Nevertheless, the detailed relationship between the seven common viruses coinfection and CAP merits in-depth investigation.

Based on our findings, future studies should be performed to address these two issues (1) elucidation of the detailed mechanisms of the interactions between viruses coinfections and other pathogens, (2) long-term and detailed follow-up of the alterations of various indexes and prognosis of various viruses coinfections with a favorable study design.

## Conclusions

Our investigation indicated that viruses coinfections were common during the course of CAP.

Multiple viruses coinfections indicated different prognosis. Different viruses coinfection yielded varying degrees of effects on the cardiac, liver, kidney and inflamatory injury in CAP. CK may be associated with the risk of viruses coinfections in CAP.

### Supplementary Information

Supplementary Material 1. Differences of various indexes among cases with different coinfected viruses.

Supplementary Material 2. Upset plot for the distribution of multi coinfection viruses.

## Data Availability

The datasets used and/or analyzed during the current study are available from the corresponding author on reasonable request.

## Abbreviations

CAP: Community-acquired pneumonia
FluA: Influenza A
FluB: Influenza B
HPIV: Human parainfluenza virus
EBV: Esptein-Barr virus
CoxsV: Coxsackie virus
CMV: Cytomegalovirus
HSV: Herpes simplex virus
WBC: White blood cell
CRP: C-reactive protein
PLT: Platelet
ESR: Erythrocyte sedimentation rate
PCT: Procalcitonin
uRBC: Urine red blood cell
BUN: Blood urea nitrogen
Scr: Serum creatinine
ALT: Alanine aminotransferase
AST: Aspartate aminotransferase
LDH: Lactate dehydrogenase
CK: Creatine kinase
CKMB: Creatine kinase isoenzyme
ROC: Receiver operating characteristic
OP: Ordinary pneumonia
SP: Severe pneumonia
SD: Standard deviation
