# Supplementary figures and images for: Coinfection of viruses in children with community-acquired pneumonia
Source: BMC Pediatr. 2024 Jul 16;24:457. doi: 10.1186/s12887-024-04939-0 (PMC11250944; doi:10.1186/s12887-024-04939-0)

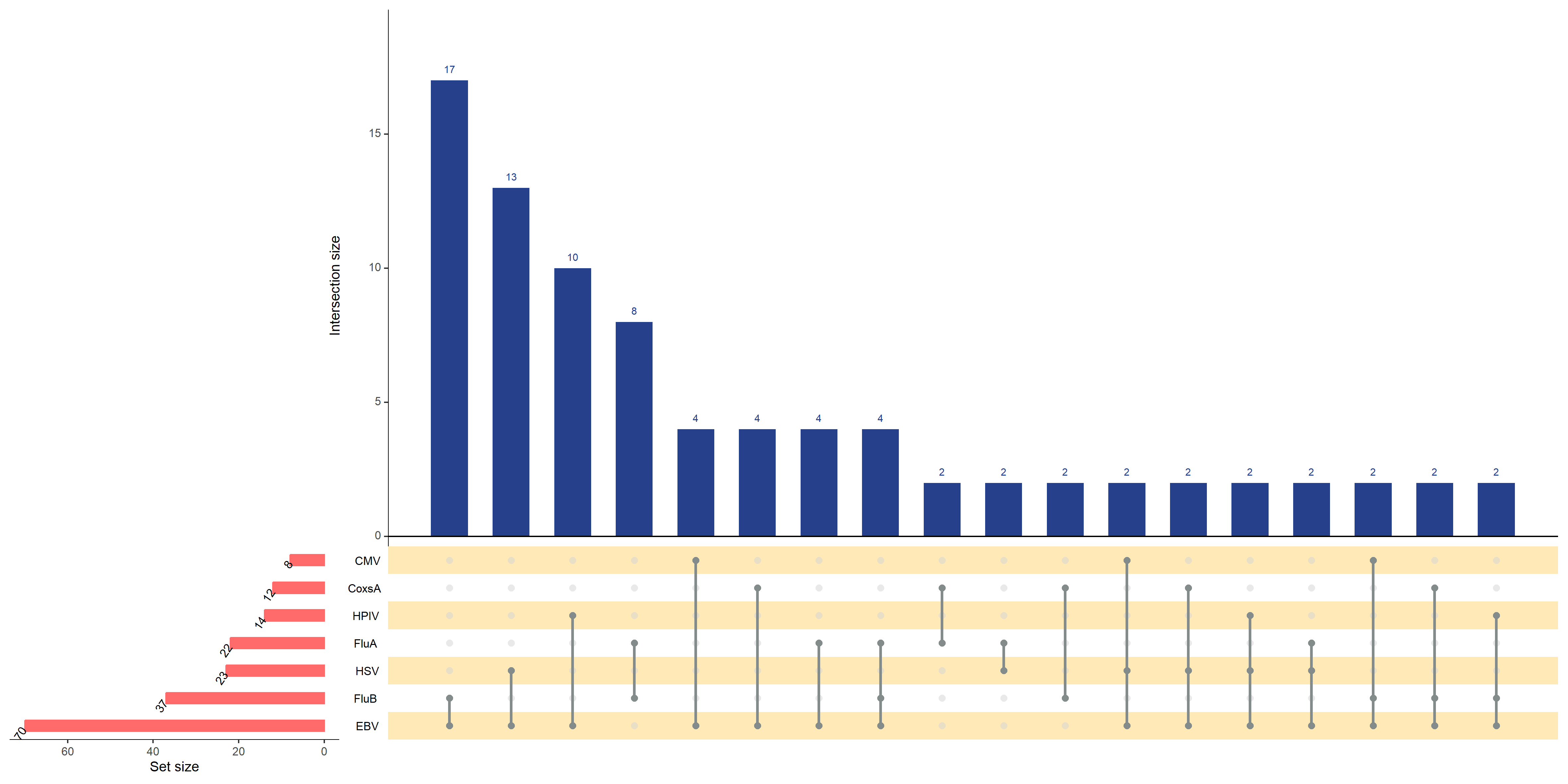

Supplement: Supplementary file 1 — Supplementary Material 1. Differences of various indexes among cases with different coinfected viruses. [file 12887_2024_4939_MOESM1_ESM.tiff]

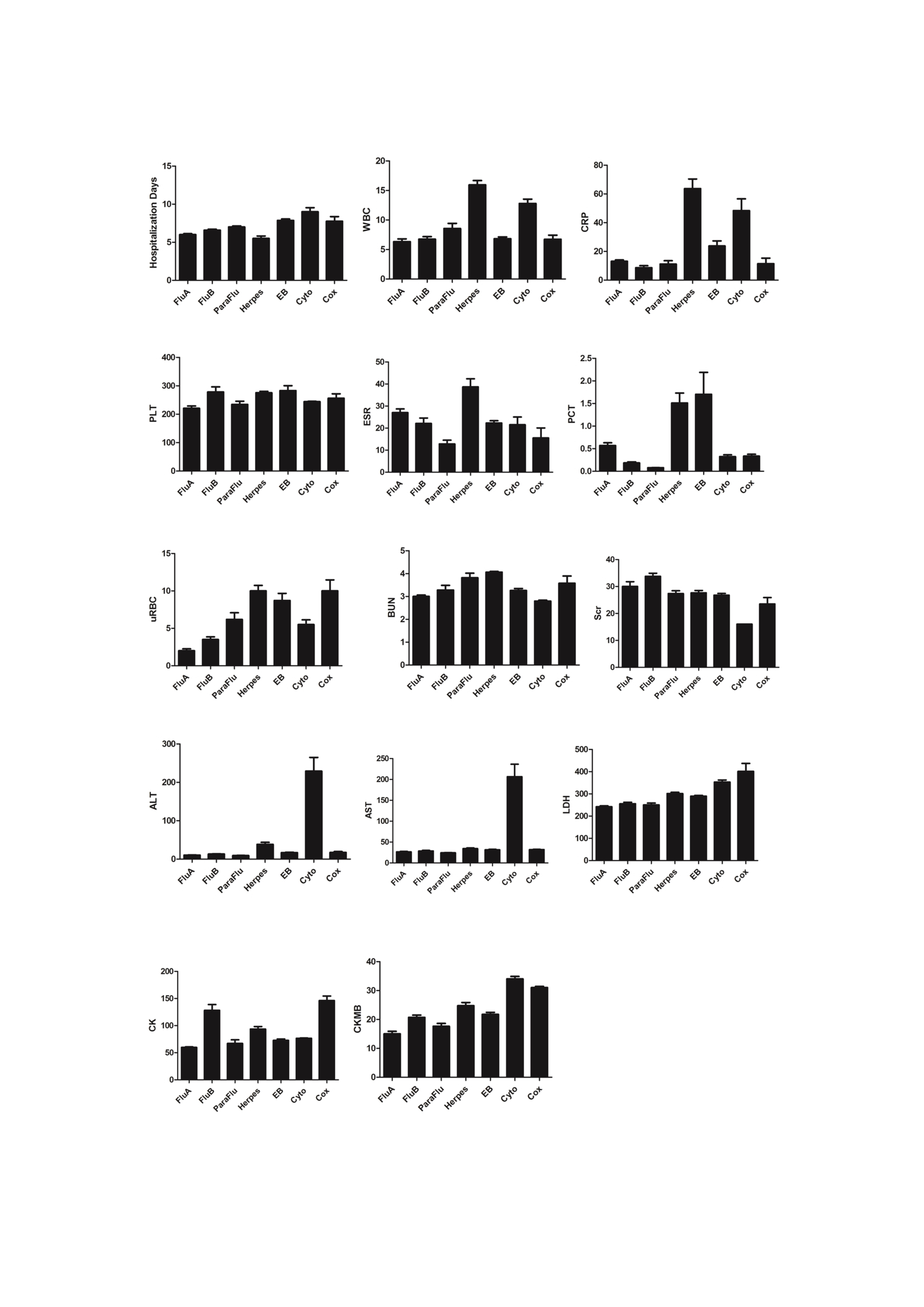

Supplement: Supplementary file 2 — Supplementary Material 2. Upset plot for the distribution of multi coinfection viruses. [file 12887_2024_4939_MOESM2_ESM.tiff]
